# Rapid Detection of Tannin Content in Wine Grapes Using Hyperspectral Technology

**DOI:** 10.3390/life14030416

**Published:** 2024-03-21

**Authors:** Peng Zhang, Qiang Wu, Yanhan Wang, Yun Huang, Min Xie, Li Fan

**Affiliations:** 1 College of Agriculture, Inner Mongolia Agricultural University, Huhhot 010010, China; 2024zhangp@gmail.com (P.Z.);; 2 Inner Mongolia Academy of Agriculture and Animal Husbandry, Huhhot 010031, China; imauwq@emails.imau.edu.cn

**Keywords:** hyperspectral, wine grape, tannin, non-destructive detection

## Abstract

Wine grape quality is influenced by the variety and growing environment, and the quality of the grapes has a significant impact on the quality of the wine. Tannins are a crucial indicator of wine grape quality, and, therefore, rapid and non-destructive methods for detecting tannin content are necessary. This study collected spectral data of Pinot Noir and Chardonnay using a geophysical spectrometer, with a focus on the 500–1800 nm spectrum. The spectra were preprocessed using Savitzky–Golay (SG), first-order differential (1D), standard normal transform (SNV), and their respective combinations. Characteristic bands were extracted through correlation analysis (PCC). Models such as partial least squares (PLS), support vector machine (SVM), random forest (RF), and one-dimensional neural network (1DCNN) were used to model tannin content. The study found that preprocessing the raw spectra improved the models’ predictive capacity. The SVM–RF model was the most effective in predicting grape tannin content, with a test set R^2^ of 0.78, an RMSE of 0.31, and an RE of 10.71%. These results provide a theoretical basis for non-destructive testing of wine grape tannin content.

## 1. Introduction

The study of phenolic compounds has become increasingly important in recent years due to their significant impact on the sensory properties of red wine [1,2]. Anthocyanins and tannins had a great influence on the quality of red wine. They influenced the organoleptic properties of wine by interacting with other components [3]. Among them, tannins are found in grape skins and seeds and are an important phenolic compound in grapes [4,5]. These compounds impart astringency to wine, which influences its taste and flavor [6]. Additionally, tannins contribute to the color changes observed during wine aging [7]. The tannin content in wine grapes increases with maturation. However, the increase in tannin content is influenced by various factors, such as grape varieties, the growth environment, and cultivation practices [8,9,10]. Therefore, monitoring tannin levels is crucial for timely adjustments in cultivation management, intervention in grape growth, and determining the optimal harvest period [11]. This process is essential for producing high-quality red wine.

Tannin extraction is predominantly conducted using chemical methods. Spectrophotometry and high-performance liquid chromatography (HPLC) represent the standard techniques for determining tannin content [12]. However, these methods are characterized by lengthy extraction cycles and cumbersome procedures [13]. Hyperspectral technology has emerged as a powerful analytical tool due to its continuous evolution. Hyperspectral technology has gained widespread use in monitoring key plant indicators to assess growth status due to its rapid and non-destructive nature [14]. Numerous researchers have utilized hyperspectral imaging to monitor grape phenotypic characteristics, soluble solids content, and anthocyanin content [15,16,17]. For instance, Zhang et al. employed hyperspectral imaging to detect tannin content in grains and established a predictive model using both full and characteristic wavelengths. The study found that hyperspectral technology enables quick and non-invasive evaluation of tannin levels in grains [18]. Maria Inês Rouxinol and her colleagues used a portable infrared spectrometer to collect spectral data on wine grapes, analyzing various components, including tannins. They then modeled the data using partial least squares regression (PLS). The results showed significant potential for accurately predicting tannin content [19].

Chen et al. used hyperspectral imaging to measure the tannin content in drying persimmons. They applied seven preprocessing methods (SG, SNV, 1D, and 2D) to prepare the data and developed models to assess the effectiveness of these methods. The results showed that SG1D and SG2D were the most effective, with R^2^ values of 0.742 and 0.857, respectively. This highlights the importance of choosing appropriate preprocessing techniques to improve model performance [20]. Gao Sheng et al. analyzed red tip berries using hyperspectral imaging and employed preprocessed hyperspectral data for modeling. They developed predictive models, such as partial least squares regression (PLS), least squares support vector machine (LSSVM), and random forest (RF), to predict the Brix and hardness of red grapes. The study found that the RF model was the most effective in predicting both Brix and hardness, with R^2^ values of 0.928 and 0.932, respectively. This highlights the importance of selecting an appropriate model to improve accuracy in predicting these parameters [21]. Julio Nogales-Bueno et al. used spectroscopic equipment to scan harvested grapes and preprocessed the hyperspectral data using multiplicative scattering correction (MSC), standardized normal variable (SNV), and detrending. The study showed that hyperspectral techniques can be used to quickly and non-destructively detect polyphenol content in grapes. It also predicted the extractable polyphenol content in red grape skins [22].

Specifically, this study focused on analyzing wine grapes from Ordos (Zhungeer Banner). Spectral data was collected using a portable geophysical spectrometer (SVC HR-1024i) and processed using various techniques, including SG, SNV, 1D, SG1D, SG1D-SNV, and RAW. The study used spectral feature extraction integrated with principal correlation analysis (PCC) to model grape tannin content. Four different estimation models were employed: random forest (RF), support vector machine (SVM), partial least squares (PLS), and 1-dimensional neural network (1DCNN). The accuracies of the models were compared to determine the most effective one for estimating tannin content.

## 2. Materials and Methods

### 2.1. Sample Preparation

**Figure 1:**
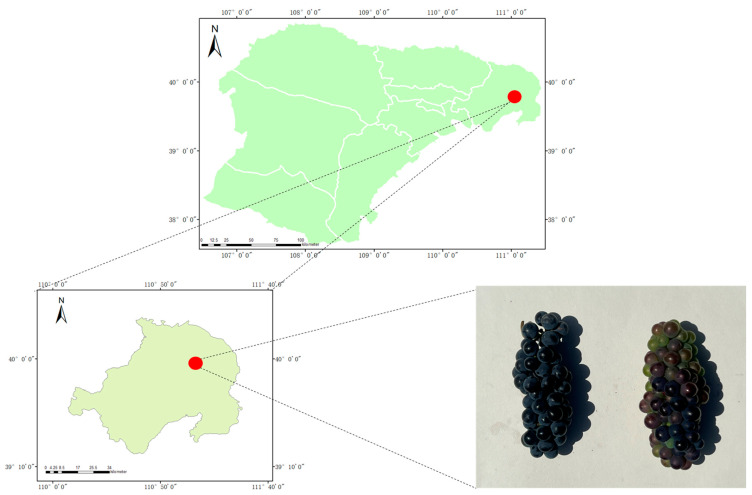
Location of the research areas and experimental designs.

The experiment was carried out in Zhungeer Banner, Ordos City, from 2021 to 2022 using Pinot Noir and Chardonnay as experimental varieties (Figure 1). Both experimental varieties were harvested at the same time. The harvest dates in 2021 were 28 August, 4 September, 11 September, and 18 September. The harvest dates in 2022 were 25 August, 1 September, 8 September, and 15 September.

### 2.2. Spectral Acquisition

In this study, a portable geophysical spectrometer (HR-1024, SVC, Manufactured by Sloan Valve Company (svc), located in Franklin Industrial Park near Chicago, IL, USA) was used with a detection range of 500–1800 nm. The built-in CPU provided data processing capacity, and the personal digital assistant (PDA) enabled real-time information transmission through remote Bluetooth technology. Prior to collecting spectral data, the instrument was calibrated by scanning a whiteboard. Data was collected by scanning the wine grape berries. During scanning, ten plants of each variety were selected. Two spikes were taken from each plant, and five grains were taken from each spike, totaling 100 grains (20 spikes). Every 10 grains were divided into a group. A total of 160 samples were collected for further analysis by making four measurements before harvesting. The spectrometer automatically adjusted the integration time according to changes in light intensity for optimal scanning. After completing the scanning process, we measured the reflectance of the grape samples using the companion software of the instrument called DARWinSP (version 1.10.8).

### 2.3. Software and Model Evaluation

The study utilized TensorFlow 2.1, a deep-learning framework, in Python (version 3.7.16). The computer was equipped with a GeForce GTX 1650 graphics card with 6 GB video memory and an Intel(R) Core (TM) i7-9750H processor operating at 2.59 GHz.

### 2.4. Measurements of Tannin Content

Tannin content was determined by the Folin–Denis method. A total of 10 g of grapes were placed in a triangular flask and 50 mL of distilled water was added. The grapes were filtered in a water bath at 60 °C for 12 h. The supernatant was extracted in a water bath at 80 °C for 20 min and filtered. A total of 2 mL of the sample filtrate was aspirated and centrifuged at 8000 r/min for 4 min and the supernatant was set aside. Then, 1 mL of 0 g/L, 20 g/L, 40 g/L, 60 g/L gallic acid standard use solution was sucked up, and 5 mL of distilled water, 1 mL of a sodium tungstate–sodium molybdate mixture and 3 mL of sodium carbonate solution were added; the concentration of gallic acid standard solution was 0 g/L, 2 g, 4 g/L, 6 g/L, respectively, and the color was developed and left for 2 h, and then 0 g/L was used as the blank of the standard curve. The absorbance was measured at 760 nm using a spectrophotometer and the standard curve was plotted. Pipette 1 mL of sample supernatant, add 5 mL of water, 1 mL of a sodium tungstate–sodium molybdate mixed solution and 3 mL of sodium carbonate solution, respectively, develop the color and leave it for 2 h, and measure the absorbance at 760 nm using the standard curve 0 g/L as blank. The tannin content of the samples was calculated as in Equation (1).

X = C × (1/V) × 250 × (1/1000) × (1/m) × 1000(1)

In this equation, ‘X’ represents the tannin content (g/L), ‘C’ is the absorbance of the sample on the standard curve (mg), ‘V’ is the volume of the test solution (mL), ‘250’ is the total volume of the extract (mL), and ‘m’ is the mass of the weighed sample (g).

### 2.5. Data Analysis Methods

#### 2.5.1. Hyperspectral Preprocessing

To enhance model accuracy, we conducted data preprocessing on the raw hyperspectral reflectance data. We employed six preprocessing methods in this experiment: SG, 1D, SNV, SG1D, SG1D-SNV and Raw. Hyperspectral preprocessing methods can reduce or eliminate the impact of unimportant data on spectral data, reduce background noise interference, and highlight spectrally valid information, thereby improving spectral sensitivity.

#### 2.5.2. Data Dimensionality

Data dimensionality reduction is a technique that eliminates redundant spectral information, reducing the likelihood of model overfitting and improving the speed of model operation. In this study, we utilized the Pearson Correlation Coefficient (PCC) method for data dimensionality reduction. The PCC method reflects the strength of the linear relationship between two variables, allowing for the screening out of characteristic bands. The calculation formula is presented below as Equation (2).

(2) $$r_{X,Y}=\frac{\mathrm{cov}\left(X,Y\right)}{\sigma_{X}\sigma_{Y}}$$

In this equation, ‘*r*’ represents the correlation coefficient, ‘*X*’ the spectral wavelength, ‘*Y*’ the grape tannin content, ‘*cov*’ the covariance, and ‘*σ*’ the standard deviation.

#### 2.5.3. Model Establishment

The data on grape tannins were split into a training set and a test set in an 8:2 ratio using Scikit-learn in Python. We developed a mathematical model for predicting grape tannin content using hyperspectral non-destructive testing and subsequently validated and evaluated the predictive ability of each model for accuracy. 

SVM is a machine learning algorithm based on the principle of structural risk minimization. It reduces the complexity of the learning machine to achieve good generalization ability while ensuring training accuracy. SVM is effective in addressing issues with small samples, nonlinearities, and high dimensions, making it widely applicable in regression problems [23]. The kernel function in SVM used the radial basis function kernel. The method to find the optimal parameters was to utilize the cross-validation method, which included the parameter *C* (penalty factor) and the parameter *δ* (variance in the RBF kernel function). In this study, the paper mentioned that *C* = 2 and *δ* = 0.7 were used as parameters for modeling.

Random forest (RF) is another machine learning algorithm proposed by Breiman in 2001, which is tailored for small-scale data. The random forest algorithm is known for its robustness and strong generalization capabilities, as well as its fast training speed. It is particularly effective in handling high-dimensional data and large-scale datasets with high accuracy [24]. This study modeled 200 decision trees, and the number of independent variables required to create branches was set to ‘auto’.

The PLS model is particularly suitable for inverse modeling of datasets with small sample sizes and is conducive to refining key spectral information. PLS merges the benefits of principal component analysis, canonical correlation analysis, and multiple linear regression. This approach offers a many-to-many linear regression model and considers the explanatory power of the independent variables for the dependent variable [25].

(3) $$y=a_{0}+a_{1}x_{1}+a_{2}x_{2}+\ldots +a_{n}x_{n}$$

where $a_{0}$ in the equation is the intercept of the regression coefficient, $a_{i}$ is the regression coefficient, $x_{i}$ are the independent variables 1 to *n*.

The 1DCNN model consists of an input module, a convolution module, a fully connected layer, and a regression output layer. The model’s input parameters include the spectral data corresponding to each sample and the measured tannin values. This model demonstrates strong generalization and nonlinear capabilities, making it suitable for the conditions and requirements of this experiment [26]. The convolutional layers of the convolutional module consisted of convolutions with 16 kernels, a size of 3 × 3, and steps of one. The number of convolutional layers was determined by the number of specially acquired features.

#### 2.5.4. Model Performance

A prediction model was developed with tannin content as the dependent variable and the model was evaluated using the coefficient of determination (R^2^) and root mean square error (RMSE). The larger R^2^ is closer to 1. It means that the model is more accurate. A smaller RMSE indicates that the accuracy of the model is more robust. The two evaluation coefficients were formulated as in Equations (4) and (5).

(4) $$R^{2}=\frac{\sum {\left({\hat{y}}_{i}-\bar{y}\right)}^{2}}{\sum {\left(y_{i}-y\right)}^{2}}$$

(5) $$RMSE=\sqrt{\frac{\sum_{i=1}^{n}{\left({\hat{y}}_{i}-y_{i}\right)}^{2}}{\dot{n}}}$$

where yi  is the actual value; $\hat{y}\mathrm{i}\;$ is the estimated value; $\bar{y}\;$ is the mean actual value of the sample; and *n* is the number of samples.

## 3. Results

### 3.1. Analysis of the Tannin Content of Grapes

Analysis of the tannin content data for the two grape varieties from the 2021–2022 harvest (Table 1) showed that the tannin content of the grapes ranged from 1.06 to 3.92, with Chardonnay having a tannin content of 1.09–3.85 and Pinot Noir having a tannin content of 1.06–3.92. In addition, in 2021 and 2022, the average tannin content of Chardonnay was 2.01 and 2.32, respectively, and the average tannin content of Pinot Noir was 2.35 and 2.66, respectively, which was higher than the average tannin content of Chardonnay in both years. 

### 3.2. Hyperspectral Data Preprocessing Analysis

**Figure 2:**
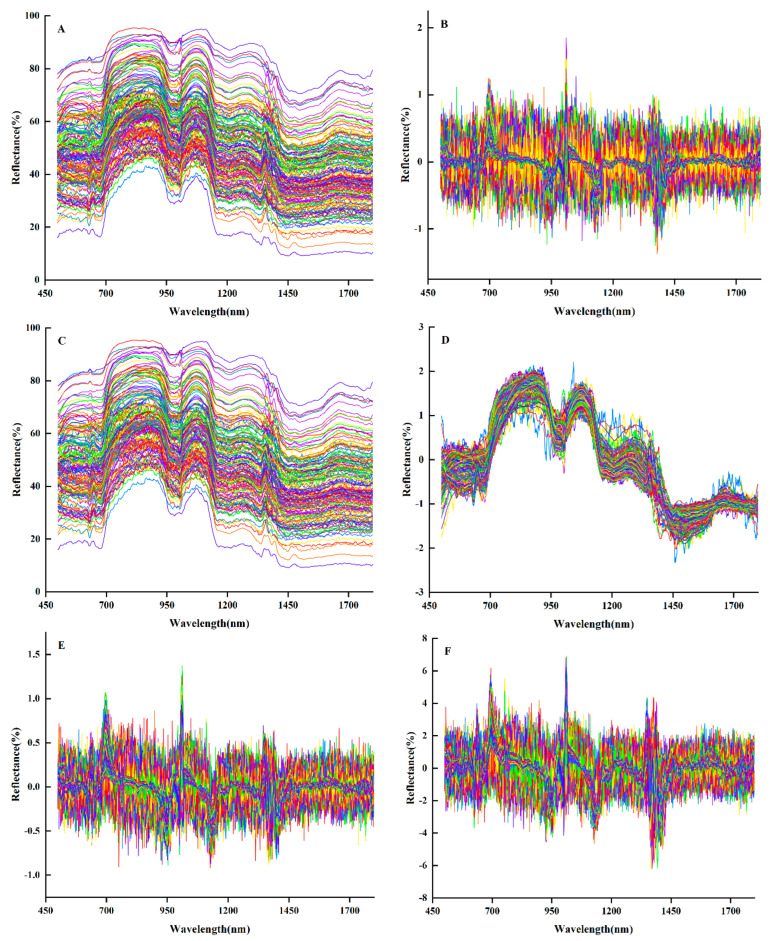
Hyperspectral image and preprocessing image. (**A**) The raw average reflectance image. (**B**) First derivative (1D) preprocessing image. (**C**) SG smoothing preprocessing image. (**D**) SNV preprocessing image. (**E**) SG1D preprocessed image. (**F**) SG1D–SNV preprocessed image.

As shown in Figure 2A, the spectral reflectance curves of grape berries showed three peaks at 920 nm, 1070 nm, and 1350 nm, which were related to the vibration of N-H and C-H groups in the samples. The troughs at 950 nm, 1130 nm, and 1400 nm were related to the C-H, N-H, and O-H of the tannins in the samples, which indicated that there was a close correlation between the spectral reflectance of tannins and the tannin content.

Given the broad range of spectral data for grape berries and the extensive number of measurement periods, the external environment can influence the spectral reflectance. The first-order derivative (1D) was selected to enhance the convergence speed of the model (Figure 2B). SG smoothing was applied for data smoothing (Figure 2C), and the standard normalized variable (SNV) was employed to eliminate the gap (Figure 2D). Furthermore, the three preprocessing methods were integrated as SG1D (Figure 2E) and SG1D-SNV (Figure 2F) for spectral data preprocessing.

### 3.3. Data Dimension Reduction

The experiment utilized the Pearson Correlation Coefficient (PCC) method to extract characteristic bands. Only bands highly correlated with grape tannins were extracted from the preprocessed data. Wavelengths with a correlation greater than 0.5 and ranking in the top 20 were used instead of the original bands. This approach reduced model complexity and shortened the modeling time. Table 2 displays the results of feature band extraction.

### 3.4. Performance of Models for Tannin Content Estimation

In this study, tannin content prediction was performed using SVM, RF, PLS, and 1DCNN with various preprocessing methods. To improve model accuracy, the dataset was divided into 128 training sets and 32 test sets, in an 8:2 ratio. Model evaluation coefficients included R^2^, RMSE and RE. The table highlights the best inversion results for each model by comparing the values of R^2^, RMSE, and RE.

#### 3.4.1. SVM Model Prediction Results

The prediction results of the SVM model were shown in Table 3, the R^2^ of the spectral training set based on 1D, SG, and SNV were all greater than 0.80, the R^2^ of the training set of the three was not much different, and the three were mainly compared from the test set R^2^. Among them, the spectral test set R^2^ = 0.77 based on SNV, and the spectral test set R^2^ of SG and 1D were 0.75 and 0.66, respectively, which were lower values than those of SNV. The test sets RMSE and RE of SG and 1D were larger than those of SNV, which indicated that, when using the SVM model to monitor the tannin content of grapes, the preprocessing of the spectra by choosing SNV could effectively improve the predictive ability and stability of the model.

#### 3.4.2. RF Model Prediction Results

The prediction results of the RF model were shown in Table 4; the R^2^ of the spectral training set with different preprocessing showed that SNV was the highest, and 1D was the second highest, with R^2^ of 0.97 and 0.96, respectively. There was not much difference in the R^2^ of the training set of the two, and the comparison was mainly drawn from the R^2^ of the test set. From the table, it could be seen that the R^2^ of the spectral test set based on SNV and 1D were 0.78 and 0.56, respectively, and the SNV spectra were better than the 1D spectra. In addition, the spectral test sets RMSE and RE of SNV were smaller than those of 1D. Therefore, when using the RF model to monitor the tannin content of grapes, choosing SNV for spectral preprocessing can effectively improve the accuracy and robustness of the model.

#### 3.4.3. PLS Model Prediction Results

The prediction results of the PLS model were shown in Table 5, the R^2^ of the spectral training sets with different preprocessing was greater than 0.5, the training set R^2^ did not differ much, and the comparison was mainly drawn from the test set R^2^. From the table, it could be seen that the test set R^2^ of 1D-based spectra was 0.69, which was much higher than the test set R^2^ of the other preprocessed spectra. In addition, the test set RMSE and RE of the spectra of 1D were 0.36 and 13.10%, which were smaller than the other preprocessed spectra. This suggested that when using the PLS model to monitor the tannin content of grapes, choosing 1D to preprocess the spectra could effectively improve the predictive ability and stability of the model.

#### 3.4.4. 1DCNN Model Prediction Results

The prediction results of the 1DCNN model were shown in Table 6; the spectral training set based on SG1DSNV had a higher R^2^ of 0.87, followed by 1D and SNV spectra with 0.79 and 0.71, respectively. From the table, it could be seen that the spectral test set based on SNV had the highest R^2^, followed by 1D and SG1D spectra with R^2^ of 0.70, 0.63, and 0.50, respectively. The other preprocessed spectral test sets all had R^2^ less than 0.5 and had poor prediction results. In addition, the spectral test sets RMSE and RE of SNV were smaller than 1D and other preprocessed spectra. This suggested that when using the 1DCNN model to monitor the tannin content of grapes, choosing SNV to preprocess the spectra could effectively improve the accuracy and robustness of the model.

#### 3.4.5. Selection of Optimal Model for Tannin Content Estimation

**Figure 3:**
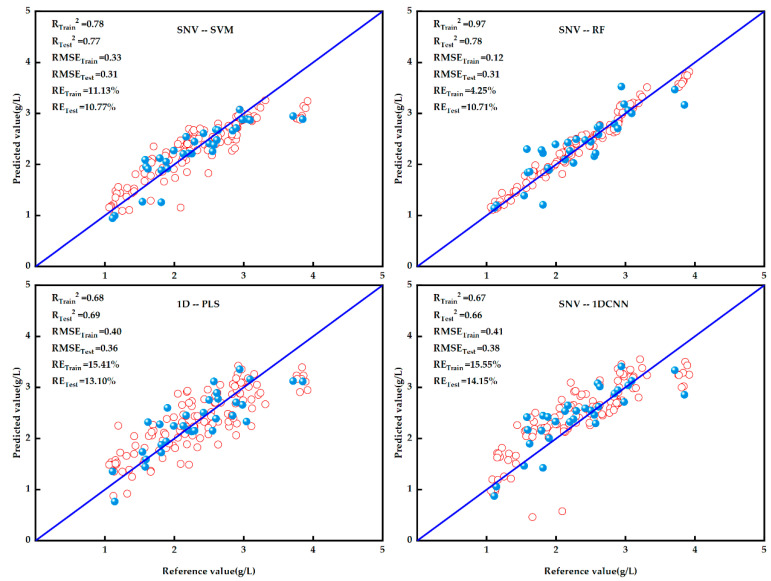
Validation results of the regression model for tannin content. Each fit is plotted for the training and test sets, with the red blobs indicating the training set data and the blue blobs indicating the test set data. The degree of model strength can be summarized based on the deviation of the model from the standard line, and these plots show R^2^, RMSE, and RE.

As depicted in Figure 3, the four modeling methods—SVM, RF, PLS, and 1DCNN— were compared. The models exhibiting the best prediction performance were selected to create independent validation scatter plots, showing both measured and predicted tannin content. The most effective predictive models were identified as SNV-SVM, SNV-RF, 1D-PLS, and SNV-1DCNN, respectively. Notably, the sample distributions in the validation and test sets of the SNV-RF model showed minimal deviation from the 1:1 line, especially when compared to those of the SNV-SVM, 1D-PLS, and SNV-1DCNN models. This distribution was essentially linear along the 1:1 line, suggesting that the prediction accuracy of the SNV-RF model surpasses that of the other three models overall. Consequently, the SNV-RF model was selected for detecting grape tannin content, as it could further enhance the accuracy and stability of the prediction results.

## 4. Discussion

In recent years, spectroscopic techniques have been widely used for the rapid monitoring of fruit substance content, among other applications [27]. Visible-near-infrared spectroscopy has been demonstrated by numerous researchers as feasible for predicting grape composition [28,29]. It is worth noting that the majority of these studies utilized raw spectral data without implementing spectral data preprocessing and feature band extraction. The use of redundant and complex spectral data resulted in decreased model prediction accuracy and operational speed. To address this issue, spectral data preprocessing is performed, followed by feature band extraction based on the preprocessed spectral data. This approach reduces the dimensionality of the spectral data and retains the feature bands that are highly correlated with the samples. Thus, this methodology effectively addresses the issues of decreased predictive ability and operational speed of the model. In this study, six preprocessing methods were used to process the spectral data, aiming to eliminate noise and enhance spectral variability, thereby improving spectral quality. Furthermore, principal component analysis (PCA) was used for feature band extraction to achieve data downscaling and simplification. The feasibility of four distinct modeling methods for the prediction of tannin content in wine grapes has also been investigated.

In this study, the raw spectral data were preprocessed, and modeling based on the preprocessed data resulted in improved model accuracy. This result is consistent with the results of the study [30]. The modeling prediction of the spectral data preprocessed by SNV was the best. This may be due to the fact that SNV standardizes and normalizes the raw data to further improve the accuracy of the spectral data and make the differences between different spectra more significant, thus enabling PCC to extract the characteristic bands more accurately. This is what leads to the higher accuracy of the model built based on the spectral data preprocessed by SNV. In addition, in this study, PCC was used for data downscaling and feature band extraction, replacing 1300 variables with 19–20 variables, which improved the running speed of the model. The results showed that the extracted feature bands were feasible for estimating grape tannin content. This is consistent with the results of the study [20]. In this study, SVM, RF, PLS and 1DCNN were used to develop an accurate quantitative model for wine grape tannin content. In this study, each of the four models was modeled based on six pre-processed spectral data, for a total of 24 combined models. The optimal models corresponding to each modeling method were SNV-SVM, SNV-RF, 1D-PLS, and SNV-1DCNN, respectively. Comparison of these four models revealed that the SNV-RF model had the strongest predictive ability, which may be due to the fact that the RF model based on SVM spectral preprocessing is more suitable for data with small sample sizes and high dimensionality of variables. The model provides a theoretical basis for the prediction of tannin content in wine grapes using small sample data.

Considering the limitations of this study, it is crucial to note that, despite examining the tannin content at the maturity of different wine grape varieties over two consecutive years, the small number of varieties selected limits the model’s generalizability. Future research could benefit from increasing the number of varieties and ecological zones, thereby enhancing the model’s generalizability. Furthermore, while PCC is employed for feature band extraction, this method may inadvertently exclude some crucial bands due to their marginally lower correlation, leading to the loss of significant bands. In subsequent research, exploring various methods for feature band extraction to achieve data dimensionality reduction and enhance model accuracy will be valuable. It should be noted that deep learning models typically demonstrate greater applicability to larger datasets. Thus, in future studies, expanding the sample size and training the model with these accumulated samples will be crucial for enhancing its predictive capability and stability.

## 5. Conclusions

In this study, we describe the complete workflow for predicting tannin content in grapes based on hyperspectral detection of tannin content in grapes, using six preprocessing methods for spectral data preprocessing. The extraction of feature bands was performed using PCC, and the data were downscaled to improve the speed of model running. And four modeling methods were used to increase the comparability of the models by pairing with the six preprocessing to determine the best model. The results show that the use of SNV for spectral data preprocessing can effectively improve the predictive ability and stability of the model when using SVM, RF and 1DCNN models for prediction. A comparison of the optimal models of the four modeling approaches revealed that the SNV-RF model had the highest accuracy and good robustness in predicting grape tannin content. Its R^2^ = 0.78, RMSE = 0.21, and RE = 10.71%. These results indicate that it is feasible to utilize hyperspectral technology for the detection of tannin content in wine grapes and to provide a theoretical basis for the rapid non-destructive detection of tannin content in wine grapes.

## Figures and Tables

**Table 1:** Statistical analysis of tannin content in two grape varieties.

Variety	Measuring Time	Sample Size	Minimum (g/L)	Maximum (g/L)	Mean (g/L)	Standard Deviation (g/L)
Chardonnay	2021	40	1.12	3.19	2.01	0.46
	2022	40	1.09	3.85	2.32	0.59
Pinot Noir	2021	40	1.06	3.31	2.35	0.70
	2022	40	1.08	3.92	2.66	0.86

**Table 2:** Extracted characteristic wavelengths.

Preprocessing Method	Number of Feature Bands	Maximum Correlation Coefficient
1D	19	0.63
SG	20	0.72
SNV	20	0.86
SG1D	19	0.60
SG1DSNV	20	0.62
RAW	20	0.73

**Table 3:** Training and testing results of SVM model to estimate tannin content.

Preprocessing Method	Train			Test		
	R^2^	RMSE	RE (%)	R^2^	RMSE	RE (%)
Raw	0.78	0.33	11.56	0.71	0.35	12.07
1D	0.80	0.31	9.63	0.66	0.38	13.61
SG	0.78	0.33	11.56	0.71	0.35	12.07
SNV	0.78	0.33	11.13	0.77	0.31	10.77
SG1D	0.64	0.43	13.90	0.43	0.49	18.39
SG1DSNV	0.59	0.45	13.86	0.37	0.52	19.53

**Table 4:** Training and testing results of RF model to estimate tannin content.

Preprocessing Method	Train			Test		
	R^2^	RMSE	RE (%)	R^2^	RMSE	RE (%)
Raw	0.96	0.14	4.72	0.80	0.29	9.64
1D	0.95	0.16	5.63	0.56	0.43	16.16
SG	0.96	0.14	4.77	0.81	0.28	9.29
SNV	0.97	0.12	4.25	0.78	0.31	10.71
SG1D	0.92	0.20	7.19	0.40	0.51	20.92
SG1DSNV	0.92	0.20	7.17	0.39	0.51	19.97

**Table 5:** Training and testing results of PLS model to estimate tannin content.

Preprocessing Method	Train			Test		
	R^2^	RMSE	RE (%)	R^2^	RMSE	RE (%)
Raw	0.67	0.41	15.07	0.57	0.43	16.66
1D	0.68	0.40	15.41	0.69	0.36	13.10
SG	0.67	0.41	15.07	0.57	0.43	16.66
SNV	0.78	0.33	11.13	0.77	0.31	10.77
SG1D	0.55	0.48	17.94	0.59	0.42	16.51
SG1DSNV	0.53	0.49	17.62	0.47	0.47	18.75

**Table 6:** Training and testing results of 1DCNN model to estimate tannin content.

Preprocessing Method	Train			Test		
	R^2^	RMSE	RE (%)	R^2^	RMSE	RE (%)
Raw	0.31	1.60	27.82	0.23	1.53	24.12
1D	0.76	0.35	10.84	0.31	0.54	21.34
SG	0.43	1.03	23.53	0.27	0.81	17.03
SNV	0.67	0.41	15.55	0.66	0.38	14.15
SG1D	0.66	0.41	14.56	0.50	0.46	19.12
SG1DSNV	0.87	0.26	8.84	0.21	0.58	23.31

## Data Availability

Limitations applied to the dataset: the data provided in this study are available upon request from the corresponding author; the data are not publicly available due to privacy.
